# Addition to “Chemical Compensation Challenges in Processing Antiferroelectric PbZrO_3_ Thin Films”

**DOI:** 10.1021/acsomega.5c12802

**Published:** 2026-01-07

**Authors:** Milan H. Haddad, Vasily Lebedev, Kristina Holsgrove, Sergio Rivera-Cruz, Sarah Stock, Nikhilesh Maity, Sergey Lisenkov, Inna Ponomareva, Amit Kumar, Lewys Jones, Nazanin Bassiri-Gharb

We would like to add to the end of the manuscript a section entitled “Data Availability” which includes the Zenodo link to the raw data.

## Data Availability

The raw data that support these findings are available at 10.5281/zenodo.15652938.

